# Approaching the Blank Document: Gamification of Manuscript Writing From a Trainee's Perspective

**DOI:** 10.1097/og9.0000000000000137

**Published:** 2026-02-19

**Authors:** Minhazur Sarker

**Affiliations:** Division of Maternal-Fetal Medicine, University of California, San Diego, La Jolla, California.

## Abstract

Eventual conversion of completed research to a written manuscript can be daunting for a trainee; gamification of the process makes it digestible and approachable.

“How do you eat an elephant? One bite at a time!”

While not literally, this advice was given to me as a naïve third-year medical student when I asked how to approach the daunting task of writing a research manuscript. My mentors recommended using daily “just 30-minute sessions.” What was intended as solid advice, for me, felt like anything but that. I spent many “just 30-minute sessions” staring at the monster that is the Blank Document, with little success. Although my mentors expressed willingness to help, I never mustered the courage to ask because I thought admitting that I had no idea what I was doing was a sign of weakness. As a result, not only did I never publish my research as a medical student, but also I never even wrote a draft. Small bites don't seem so easy if you can't find anywhere to start chewing!

Although my experience is my own, a similar reflection is not uncommon among trainees in our field. Not surprisingly, this may contribute to the lack of conversion of conference abstracts to manuscript publication historically noted within the Society of Gynecologic Oncology and the Society for Maternal-Fetal Medicine.^1–3^ Earlier this year, I published an updated analysis of manuscript conversion across multiple obstetrics and gynecology subspecialty conferences, noting that despite contemporary improvements in dissemination pathways and more journals to choose from, our conversion rates remain poor.^4^

Almost a decade since I began, writing is still not my strong suit. However, I have conquered the Blank Document and experienced the joy of seeing my manuscripts in journals many times. I have learned a thing or 10 about the Blank Document's strengths and weaknesses. My struggles have given rise to an enthusiasm for sharing my approach with mentees and their mentors, and I believe gamification is the solution. By creating a game with storyline chapters and points for each battle, I have created a means to make this daunting task fun and feasible. My hope is that those who are newly grappling with the art of manuscript writing will find this approach useful and that those more seasoned can use it as a framework to guide mentees.

Download Appendix 1 (available online at http://links.lww.com/AOG/E432) and follow along (or refer to Fig. 1). The short text here is meant to serve as a lighthearted tactical plan for any trainee asked to join the battle toward a clinical research manuscript. Please take what you find useful and reject what doesn't work for you (although please do tell me if you find it helpful because who doesn't like a good pat on the back).

**Fig. 1.**
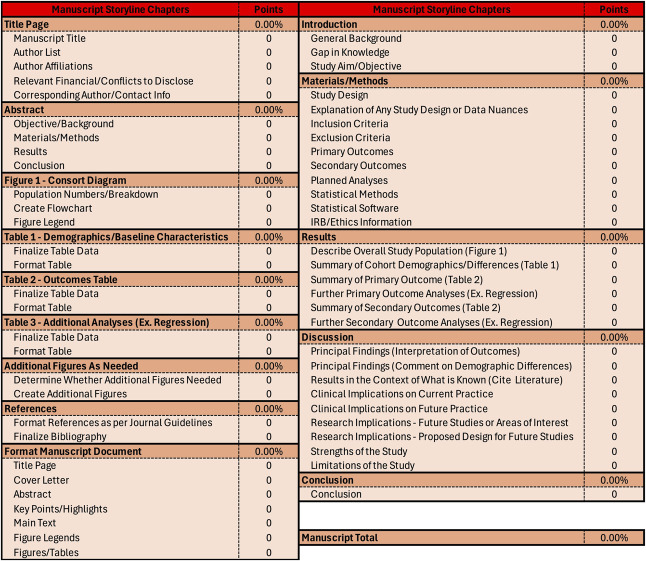
Blank Document overall battle plan.

*Sarker. Manuscript Writing From a Trainee’s Perspective. Obstet Gynecol 2026*.

## TECHNIQUE

### Gearing Up

Of course, one cannot just waltz into battle without the right equipment, so I'll take a moment to discuss gearing up. This involves a few quick, but key, steps. Once you've completed the preparations, onward!

- Step One: Analyze the data. Be sure to discuss the findings and their interpretation with your research team. Nothing is worse than the inefficiency of creating a draft that is interpreted incorrectly.
- Step Two: Gather the relevant literature. I recommend this be compiled and summarized in a separate document. During this process, it is worthwhile to save each reference in your favorite citation manager (I recommend checking with your peers or mentors for their preferred tool).
- Step Three: Determine your target journal. This helps you format it in accordance with the author guidelines.
- Step Four: Create a bulleted outline of the major topics and points you wish to cover (also a nice spot to note references for each of these points).

“Just start writing” …by attacking the minions first

**Fig. 2.**
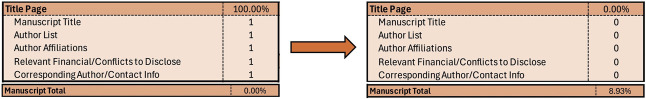
Conquering the title page chapter.

Despite the ubiquity of the “just start writing” command, it is one of the hardest tasks for an inexperienced scientific writer. But you know what *is* easy to start writing? The manuscript title, your author list, author affiliations, relevant financial conflicts to disclose, and the corresponding author's contact information. Remember Appendix 1 (available online at http://links.lww.com/AOG/E432)? Navigate to the “Title Page” section. Each component is worth 1 point, so change every “0” to “1.” The form will autoupdate, and just like that, you have the title page, your first manuscript page, and your first storyline chapter completed. Look at the “Manuscript Total” in the bottom right for your dopamine hit, seeing that you're almost 10% finished (Fig. 2). That wasn't so bad!

*Sarker. Manuscript Writing From a Trainee’s Perspective. Obstet Gynecol 2026*.

### Pretty Pictures and Boxes

**Fig. 3.**
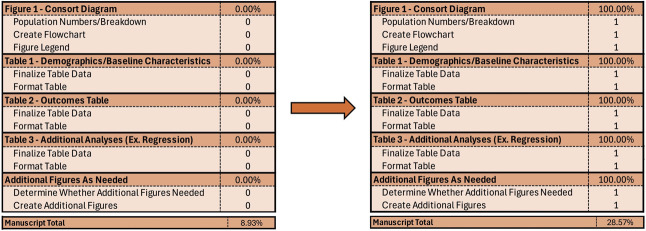
Finalizing figures and tables.

After tackling that first page and enjoying the fruits of my labor, I like to attack the figures and tables. This step is relatively straightforward but extremely important because, given most people’s busy daily lives, figures and tables are more digestible for the reader than the text. Often, this is already roughly completed having done Step One of gearing up. Simply finalize them one at a time, and now you can copy and paste them into the Blank Document (I often use Microsoft Excel for figures and tables, but there are many other options, including statistical software or editing programs—again, I recommend checking with your team or mentors for their preferred tool). Navigate to the Figures and Tables section of Appendix 1 (available online at http://links.lww.com/AOG/E432) and drop some 1s into the cells. With these steps, you've completed another few storyline chapters, and you're almost at 30% complete (Fig. 3). Crushing it.

Okay—We Need Some Words…

*Sarker. Manuscript Writing From a Trainee’s Perspective. Obstet Gynecol 2026*.

Listen, I am not delusional—I realize we've avoided the actual writing to focus on the other tasks, but I can tell you that nearly 30% completion is nearly 30% completion. The rush that is achieved by seeing the Blank Document partially defeated will provide much-needed momentum when the rubber hits the road during actual writing.

The manuscript writing storyline chapters are located on the right side of the point sheet, and each section is broken down into component pieces. Each of these components? You guessed it—worth separate points.

**Fig. 4.**
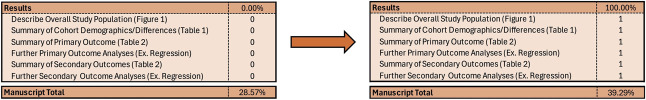
Completing the results section.

**Fig. 5.**
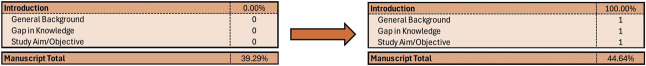
Framing the “why” (introduction).

**Fig. 6.**
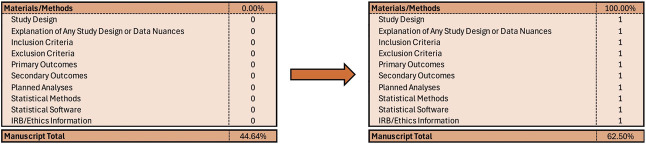
Describing the materials and methods.

At this point, this becomes a choose-your-own adventure, and you pick what you believe is more manageable. Having just created the figures and tables, I often describe them in words using the document prompts and BOOM—results section complete (Fig. 4). Summarize why you or anyone should even care about your planned work and KABOOM—introduction complete (Fig. 5). Subsequently, talk about what directions you followed and what you did to get to your results and BAM—materials and methods section (Fig. 6) complete. Notably, while writing a methods section may appear challenging, tackling one objective at a time (eg, study time periods, listing inclusion and exclusion criteria, or defining primary and secondary outcomes) is much more approachable.

*Sarker. Manuscript Writing From a Trainee’s Perspective. Obstet Gynecol 2026*.

*Sarker. Manuscript Writing From a Trainee’s Perspective. Obstet Gynecol 2026*.

*Sarker. Manuscript Writing From a Trainee’s Perspective. Obstet Gynecol 2026*.

### The Meat and Potatoes of the Paper

**Fig. 7.**
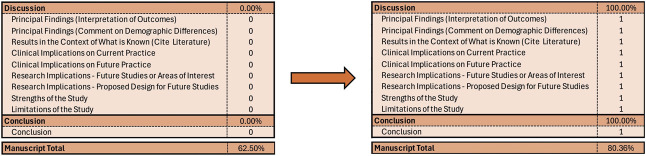
Interpreting and contextualizing the findings.

The component of the manuscript that often creates the greatest strain and unease, even for seasoned writers, is the discussion section. This section is critical to explaining the interpretation of your findings and their implications on clinical and research endeavors. Moreover, it provides the writer the opportunity to defend their strengths and limitations. Although this section often benefits most from the revisions that come after the first draft, the subsections remain formulaic (Fig. 7). This portion of the manuscript may take a bit more time to write, so sometimes I formulate my thoughts over time and revisit for numerous passes. Finally, a succinct conclusion section leaves the reader walking away with your most important message.

*Sarker. Manuscript Writing From a Trainee’s Perspective. Obstet Gynecol 2026*.

### Final Steps

**Fig. 8.**
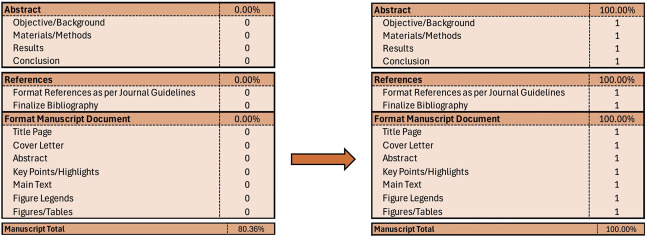
The much anticipated 100%!

Once you finish the game, you have a crude imperfect product. That is the goal! Now your mentors can take it from here to refine this now not-so-Blank Document (even this document had help—see the acknowledgments section)! After the revisions have been well underway, I look toward writing the abstract. Keep in mind that sometimes (probably often), many people will read only the abstract of your manuscript. This piece of your entire manuscript, although short, is your “sales” and “elevator” pitch, so to speak, and thus, is critical. Writing the abstract at the end of your journey often is your best bet! Finally, I format the references and document to the journal's specifications. Once all of these are complete, you reach the much anticipated 100% completion (Fig. 8).

*Sarker. Manuscript Writing From a Trainee’s Perspective. Obstet Gynecol 2026*.

### How to Use the Battle Plan

Open the document and “Save As” with a title that fits your manuscript. When you complete a subsection, simply change the “0” to a “1,” and all the storyline chapter summaries and manuscript totals will automatically update. Although this battle plan is written for a research manuscript on a cohort study, it can be modified easily for other study designs and other journals as needed.

## EXPERIENCE

If you review the objectives listed within each writing storyline chapter, you'll find that they mirror the bulk of the components found in most clinical research manuscripts. This is by design, because manuscript writing is formulaic, and using this gamified system can help one adopt this formula into habit.

Despite starting my training in medicine with a phobia of writing, I now gear up for battle systematically to ensure that each and every time I confront the Blank Document, I am as prepared as possible. The actual fight with the beast is methodical, formulaic, and efficient. Honestly, I no longer need the assistance of the gamified system. Take for example this piece written here; achieved in one sitting, tackled head on, and without any fear or hesitation. The Blank Document has been slayed. Dare I say—crushed it.

Beyond my own personal use of the technique, I have used it to mentor the medical students and resident physicians that work with me on various research projects. I start this process by sending a detailed email summarizing the journey we've taken. Then I create a shared web-based, online Blank Document (typically Microsoft Excel or Google Sheets) for each manuscript in progress. I notify the mentee that this document is a way for us, as mentors, to monitor their progress and assist them along the way with specific subsections. Although I have only experienced doing so with a few mentees thus far, each response has been favorable toward the technique.

My intention is that the technique described here will create a safe and approachable method for members of the scientific community newly tackling the daunting process of manuscript writing. Whether it be from this technique or another manner, I would love to see increased dissemination of the amazing work being conducted in the field of obstetrics and gynecology. My greatest accomplishment would be to help even one lost soul through this journey. If I can do it, so can you. Give it a shot!
